# Dysfunction of Inflammatory Pathways and Their Relationship With Psychological Factors in Adult Female Patients With Eating Disorders

**DOI:** 10.3389/fphar.2022.846172

**Published:** 2022-04-19

**Authors:** Javier R. Caso, Karina S. MacDowell, Marta Soto, Francisco Ruiz-Guerrero, Álvaro Carrasco-Díaz, Juan C. Leza, José L. Carrasco, Marina Díaz-Marsá

**Affiliations:** ^1^ Departamento de Farmacología y Toxicología, Facultad de Medicina, Instituto de Investigación Sanitaria Hospital 12 de Octubre (Imas12), Instituto Universitario de Investigación en Neuroquímica UCM, Universidad Complutense de Madrid, Madrid, Spain; ^2^ Centro de Investigación Biomédica en Red de Salud Mental (CIBERSAM), Madrid, Spain; ^3^ Departamento de Medicina Legal, Psiquiatría y Patología, Facultad de Medicina, Universidad Complutense de Madrid, Madrid, Spain; ^4^ Instituto de Investigación Sanitaria, Hospital Clínico San Carlos, Madrid, Spain; ^5^ Hospital Universitario Marqués de Valdecilla, Santander, Spain; ^6^ Facultad de Educación y Psicología, Universidad Francisco de Vitoria, Madrid, Spain

**Keywords:** eating disorders, affective disorders, impulsivity, depressive symptomatology, cytokines

## Abstract

The attempts to clarify the origin of eating disorders (ED) have not been completely successful and their etiopathogenesis remains unknown. Current research shows an activation of the immune response in neuropsychiatric diseases, including ED. We aimed to investigate immune response parameters in patients with ED and to identify psychological factors influencing the inflammatory response. The relationship between inflammation markers and impulsivity and affective symptomatology was explored as well. Thirty-four adult female patients with current diagnosis of ED, none of them under psychopharmacological treatment (excluding benzodiazepines), were included in this study. Patients were compared with a healthy control group of fifteen adult females. The levels of inflammatory markers and indicators of oxidative/nitrosative stress were evaluated in plasma and/or in peripheral blood mononuclear cells (PBMCs). Subjects were assessed by means of different ED evaluation tools. Additionally, the Barratt Impulsiveness Scale, the Montgomery-Asberg Depression Rating Scale and the Hamilton Anxiety Rating Scale were also employed. Patients with ED shown increased plasma levels of the pro-inflammatory nuclear factor *kappa* B (NFκB) and the cytokine tumor necrosis factor-*alpha* (TNF-α), among other factors and an increment in the oxidative/nitrosative stress as well as increased glucocorticoid receptor (GR) expression levels in their PBMCs. Moreover, the inflammatory prostaglandin E_2_ (PGE_2_) correlated with impulsiveness and the anti-inflammatory prostaglandin J_2_ (15d-PGJ_2_) correlated with depressive symptomatology. Our results point towards a relationship between the immune response and impulsiveness and between the immune response and depressive symptomatology in female adult patients with ED.

## Introduction

Eating disorders (ED) are a group of mental conditions including, among others, Anorexia Nervosa (AN), Bulimia Nervosa (BN), and Unspecified Feeding or Eating Disorder (American Psychiatric Association, 2013). These conditions represent a public health matter not only because of life prevalence of AN (Lindvall Dahlgren et al., 2017) but also for the terrific suffering that they cause in patients and their consequences, in some cases, even death.

Efforts to clarify the origin of ED have not been completely successful to the date. Some psychological factors, sociocultural influences, intergenerational effects, and biological and genetic predispositions have been proposed as risk factors for these disorders (Culbert et al., 2015) but their etiopathogenesis remains unknown.

Among possible biological causes, changes in several neurotransmitters (Grzelak et al., 2017) or genetic components (Scherag et al., 2010; Bulik et al., 2016) have been proposed. Similarly, immuno-endocrine factors have been related with ED (Brambilla et al., 2001). Relationships between hyper-reactivity to stress and raises in cortisol levels have been observed (Monteleone et al., 2014) as well as a linkage between stress response and inflammation (Paszynska et al., 2016). Hypercortisolemia steers to the production and gathering of cytotoxic proinflammatory parameters in the peripheral nervous system (PNS) and in the central nervous system (CNS) (Sorrells et al., 2009).

Attempts on the recognition of potential biomarkers suggest variations in the proinflammatory cytokines expression in patients with ED (Corcos et al., 2003; Dalton et al., 2018). Previous studies have also reported increased levels of pro-inflammatory cytokines such as tumor necrosis factor-*alpha* (TNF-α), interleukin (IL)-1*beta* (IL-1β) and IL-6 in patients with ED (Nova et al., 2002; Kahl et al., 2004; Ahren-Moonga et al., 2011; Macdowell et al., 2013).

Inflammation is controlled by inter- and intracellular processes. Analyses have indicated a stimulation of a number of elements of the immune response [i.e., the nuclear factor *kappa* B (NFκB), the inducible isoform of the nitric oxide synthase (iNOS) and the inducible cyclooxygenase-2 (COX-2)] in rodent models and/or in samples from patients with psychiatric conditions (Dantzer et al., 2008; Garcia-Bueno et al., 2008; Leonard and Maes, 2012). Interestingly, COX-2 and the final markers of lipid peroxidation of the cell membranes have been observed to be augmented in plasma from adult ED patients (Macdowell et al., 2013).

COX-2 also participates in the compensatory anti-inflammatory response. Amongst the most significant anti-inflammatory processes are the cyclopentenone prostaglandins produced from the COX-2 activation by different pathophysiological elements, and the activities of the *gamma* isoform of the peroxisome proliferator-activated receptor (PPARγ) (Kapadia et al., 2008; Popa-Wagner et al., 2013).

Several stress-related neuropsychiatric diseases, including ED, have been related to the dysfunction of both pro- and anti-inflammatory pathways and to an increase in inflammation (Garcia-Bueno et al., 2008; Dunjic-Kostic et al., 2013). Even more, inflammation has been identified as a differential factor in ED subtypes (Anderson et al., 2018).

Some clinical aspects of psychiatric disorders like impulsiveness are related to an impairment in the inflammatory mechanisms (Sutin et al., 2012) and the relationship between impulsivity and ED has been corroborated (Waxman, 2009), including in BN (Merlotti et al., 2013) and in AN (Lavender et al., 2017).

Affective symptoms are comorbid with ED (Hudson et al., 2007) and higher levels of depression and general anxiety correlate with higher ED symptomatology (Smith et al., 2018); therefore, these aspects can determine the evolution of ED, as well as their prognosis and treatment (Vall and Wade, 2015).

The aim of this study was to examine pro/anti-inflammatory parameters and related risk pathways in patients with ED. Usually, studies focus on adolescent patients. We wanted to study a group of patients that we do believe is underrepresented in the usual studies: adult females and with a long history fighting with the disease. That was the reason for choosing a specific age range and gender. Thus, a group of adult female patients with ED (none of them under psychopharmacological treatment, excluding benzodiazepines) and a healthy control group were compared.

Consequently, several immune parameters implicated in the regulation of inflammation and in the pro/anti-inflammatory balance, including the resulting oxidative/nitrosative factors, were studied in plasma and/or peripheral blood mononuclear cells (PBMCs). The choice of PBMCs is founded in their actions providing selective responses to the immune system and in being major cells in the human body immunity as well as their plausible role as a source of inflammatory biomarkers.

Finally, the relationship between inflammation markers and impulsivity and affective symptomatology was explored as well, aiming to identify psychological factors that might potentially influence the inflammatory response in ED.

## Material and Methods

### Sample and Clinical/Psychological Tests

The criteria of DSM-IV-TR were used for diagnosis (American Psychiatric Association, 2000). Thirty-four female patients with present diagnosis of ED were included in this study: 11 of them were diagnosed with anorexia nervosa (AN), from which 8 had a diagnosis of AN restricting type -ANr- and three had a diagnosis of AN purging type -ANp-, nine had a diagnosis of bulimia nervosa (BN) and 14 had a diagnosis of Not Otherwise Specified (EDNOS). Patients were recruited at the Eating Disorders Unit of a general hospital (*Hospital Clínico San Carlos*, Madrid, Spain) and were evaluated by a senior psychiatrist who was responsible for the process of diagnosis. All subjects were outpatients and none of them was under psychopharmacological treatment (excluding benzodiazepines). To depict the psychopathology of the condition, patients were assessed by means of different ED evaluation tools comprising the Eating Disorders Inventory (EDI) (Garner et al., 1983), the Body Shape Questionnaire (BSQ) (Cooper et al., 1987), and the Bulimic Investigatory Test Edinburgh (BITE) (Henderson and Freeman, 1987). In addition, the Barratt Impulsiveness Scale (Patton et al., 1995) was employed to assess impulsiveness. Patients also completed the Montgomery-Asberg Depression Rating Scale (MADRS) (Montgomery and Asberg, 1979) and the Hamilton Anxiety Rating Scale (Hamilton, 1960).

Inclusion criteria for patients were: 1) aged 18–45 years; 2) Diagnose of Eating Disorder according to DSM-IV-TR criteria and evaluated by an expert psychiatrist. Exclusion criteria were: 1) severe physical conditions, such as organic brain syndrome or neurological disease that could affect neuropsychological performance; 2) Intelligence Quotient IQ < 85; 3) Major Depression Disorder (MDD) or substance misuse within the last 6 months; and 4) DSM-IV-TR criteria for schizophrenia, severe psychotic disorder or bipolar disorder.

The control group included 15 females which did not present any other current psychiatric medical disorder that could potentially affect inflammatory parameters. Controls were assessed by a psychologist and, in addition to present axis I disorders such as major depression, dysthymia or substance dependence disorders, lifetime history of schizophreniform or bipolar disorder were also counted as exclusion criteria for the research. Inclusion criteria for controls were: 1) aged 18–45 years old; and 2) matched in age, sex, and educational level with patients. Exclusion criteria for controls were the same that for patient, in addition to do not meet full or subthreshold criteria for ED, either restrictive or bulimic types.

No participants had fever or any allergies, ongoing infections, or other serious physical conditions at the time of assessment, and they had not received immunosuppressive drugs or vaccines for at least 6 months or anti-inflammatory drugs for at least 2 days before blood sampling. Ethical approval was obtained from the *Hospital Clínico San Carlos* Ethics Committee. All participants signed written informed consent after receiving a complete description of the study.

### Specimen Collection and Preparation

If not acknowledged, the chemicals and reagents utilized were provided by Sigma-Aldrich (Spain).

Venous blood samples (10 ml) were collected between 8:00 and 10:00 h after overnight fasting. Samples were kept at 4°C until preparation after approximately 1 h. Blood tubes were centrifuged (641 g × 10 min, 4°C). The resultant plasma samples were collected and stored at −80°C. The rest of the sample was 1:2 diluted in culture medium (RPMI 1640, LifeTech) and a gradient with Ficoll-Paque (GE Healthcare) was used to isolate mononuclear cells by centrifugation (800 g × 40 min, room temperature–RT-). The PBMC layer was aspired, re-suspended in RPMI and centrifuged (1,116 g, 10 min, room temperature). The supernatant was removed, and the mononuclear cell-enriched pellet was stored at −80°C.

### Determinations in Plasma

#### Cytokine Levels

Enzyme immunoassays (EIA) kits (Cayman Europe, Estonia) adhering to the manufacturer’s instructions were employed to measure the TNF-α and IL-1β plasma levels.

#### Prostaglandin Levels

Commercially available EIA kits (Enzo, Switzerland) were used to measure the prostaglandin (PG) E_2_ and 15-deoxy-∆^12,14^- PGJ_2_ (15d-PGJ_2_) plasma levels.

#### Lipid Peroxidation

It was assessed by Thiobarbituric Acid Reactive Substances (TBARS) assay (Cayman Europe, Estonia) following the manufacturer’s instructions.

### Measurements in PBMCs

#### Preparation of Nuclear and Cytosolic Extracts From PBMCs

PBMC samples were first fractionated in nuclear and cytosolic extracts using a procedure extensively utilized which delivers a high purity nuclear extract, almost without cytosolic residue (Garcia-Bueno et al., 2014; Caso et al., 2020).

#### Western Blot Analysis

The protein levels of the nuclear and cytosolic extracts were adjusted and then mixed with Laemmli sample buffer combined with β-mercaptoethanol (Bio-Rad, Hercules, CA). Then, samples were protein-size split in 10% SDS-polyacrylamide gel electrophoresis (90 V). Proteins from the gels were blotted onto a nitrocellulose membrane with a semi-dry transfer system (Bio-Rad). After the gel electrophoresis the membranes were blocked in 30 ml Tris-buffered saline containing 0.1% Tween 20 and 5% skim milk/BSA and were incubated with specific antibodies. The proteins to analyze were chosen based in a previous study, as well as the antibodies and their dilutions (Caso et al., 2020): 1) iNOS (Santa Cruz Biotechnology Cat# sc-650, RRID:AB_631831, diluted 1:750); 2) COX-2 (Santa Cruz Biotechnology Cat# sc-1747, RRID:AB_2084976, diluted 1:1,000); 3) PPARγ (Santa Cruz Biotechnology Cat# sc-7196, RRID:AB_654710, diluted 1:1,000); 4) phospho-p38 (Santa Cruz Biotechnology Cat# sc-17852-R, RRID:AB_2139810, diluted 1:750); 5) p38 (Santa Cruz Biotechnology Cat# sc-7972, RRID:AB_628079, diluted 1:750); 6) phospho-ERK (Cell Signaling Technology Cat# 8544, RRID:AB_11127856, diluted 1:1,000); 7) ERK (Cell Signaling Technology Cat# 4695, RRID:AB_390779, diluted 1:2,000); 8) NFκB p65 (Santa Cruz Biotechnology Cat# sc-372, RRID:AB_632037, diluted 1:1,000); 9) GR (Santa Cruz Biotechnology Cat# sc-1004, RRID:AB_2155786, diluted 1:1,000); 10) β-actin (Sigma-Aldrich Cat# A5441, RRID:AB_476744, diluted 1:10,000); 11) GAPDH (Sigma-Aldrich Cat# G8795, RRID:AB_1078991, diluted 1:5,000).

After washing with a TBS-Tween solution, the membranes were incubated with the respective horseradish peroxidase-conjugated secondary antibodies for 90 min at room temperature and revealed by ECL™-kit (Amersham Ibérica, Spain).

Blots were imaged utilizing an Odyssey^®^ Fc System (Li-COR Biosciences) and quantified by densitometry (NIH ImageJ^®^ software, RRID:SCR_003070). All measures are stated in arbitrary units of optical density (O.D.). Various exposition times were analyzed to guarantee the linearity in the intensity of the bands. The β-actin and the GAPDH were used as loading controls for the cytosolic fraction and the nuclear fraction, respectively.

#### Protein Levels

Protein levels were determined employing the method of Bradford, founded on the principle of protein-dye binding.

#### Statistical Studies

Data are expressed as mean ± SEM. Biochemical data were analyzed using the D’Agostino and Pearson normality test to assess Gaussian distribution, followed by an unpaired two-tailed *t* test. When data did not follow a Gaussian distribution, a Mann-Whitney test was performed. The Grubb’s test/extreme studentized deviate method (ESD) was performed with a significance level set at 0.05 for the detection of outliers. For the study of the clinical characteristics data were analyzed using the D’Agostino and Pearson normality test to assess Gaussian distribution, followed by an unpaired two-tailed *t* test. When data did not follow a Gaussian distribution, a Mann-Whitney test was performed. When the *F*-test indicated that variances were significantly different, an unpaired two-samples *t*-test with Welch’s correction was performed. Data were analyzed with the GraphPad Prism (v 7.00) software. As previously described (Caso et al., 2020) the association among inflammation biomarkers and clinical variables, measured by the psychometric tests, was evaluated by means of the Pearson correlation coefficient for all the variables except factors of ED: drive for thinness, Bulimic Symptomatology, Body dissatisfaction, Ineffectiveness and low self-esteem, perfectionism, interpersonal distrust, interoceptive awareness and maturity fears. This evaluation was made with SPSS (v.21). In all cases, a *p* value <0.05 was considered statistically significant.

## Results

### Clinical Characteristics of Patients and Controls

In patients, the mean duration of the illness was 12.23 (±9.8) years. The sample mean age was 28.56 (±8.5) years old. Mean scores for ED and for impulsiveness are displayed in Table 1. The mean value of the body Mass Index (BMI) was 21.15 ± 7.27 kg/m^2^.

**TABLE 1:** Mean tests scores for ED and for anxiety, impulsiveness, and depressive symptomatology.

		Patients with ED Mean (SD)	Control Mean (SD)
BMI		21.15 (7.27)	21.79 (3.81)
BIS-11	*Global Score*	45.72 (16.5)	43.64 (14.47)
	*Attentional impulsiveness*	14.91 (7.34)	12.55 (4.7)
	*Motor impulsiveness*	16.25 (8.49)	13.86 (4.8)
	*Non planning impulsiveness*	15.50 (7.86)	12.55 (4.9)
BITE	*Global Score*	29.23 (16.18)***	3.33 (3.22)
	*Symptoms*	17.08 (8.85)***	2.50 (2.84)
	*Severity of illness*	11.54 (8.23)***	0.67 (0.88)
EDI	*Global Score*	74.29 (45.59)***	9.36 (8.59)
	*Drive of thinness*	12.50 (6.34)**	6.92 (7.24)
	*Bulimic symptomatology*	6.92 (7.20)***	0.33 (0.88)
	*Body dissatisfaction*	17.67 (8.53)***	2.42 (3.55)
	*Ineffectiveness and low self-esteem*	14.08 (8.66)***	0.75 (1.42)
	*Perfectionism*	6.50 (4.40)***	2.33 (2.57)
	*Interpersonal distrust*	6.17 (4.84)***	1.25 (1.13)
	*Interoceptive awareness*	13.00 (7.82)***	1.50 (2.19)
	*Maturity Fears*	10.67 (6.89)***	1.25 (1.28)
BSQ	*Global Score*	140.79 (46.76)***	56.17 (32.59)
	*Body dissatisfaction*	77.00 (26.53)***	30.17 (18.11)
	*Weight concern*	63.79 (20.61)***	31.00 (18.82)
HARS	*Global Score*	27.64 (12.91)***	3.50 (4.8)
MADRS	*Global Score*	23.73 (12.49)***	3.36 (5.44)

Different psychological evaluation tools employed: the Barratt Impulsiveness Scale (BIS), the Bulimic Investigatory Test Edinburgh (BITE), the Eating Disorders Inventory (EDI), the Body Shape Questionnaire (BSQ), the Hamilton Anxiety Rating Scale (HARS) and the Montgomery-Asberg Depression Rating Scale (MADRS). Data are shown as Mean (Standard Deviation–SD-). Data were analyzed using the D’Agostino and Pearson normality test to assess Gaussian distribution, followed by an unpaired two-tailed *t* test. When data did not follow a Gaussian distribution, a Mann-Whitney test was performed. When the *F*-test indicated that variances were significantly different, an unpaired two-samples *t*-test with Welch’s correction was performed.

A *p* < 0.05 was considered statistically significant; **p < 0.01, ***p < 0.001 *vs.* Control.

The control group mean age was 22.53 (±2.5) years. Members of this group did not present any other current psychiatric medical disorder that could potentially affect inflammatory parameters. Mean BMI was 21.79 (±3.81) kg/m^2^.

The clinical characteristics and the comparisons between controls and patients can be seen in Table 1.

### ED Affect Plasma Levels of Pro-Inflammatory Cytokines and Oxidative/Nitrosative Components

**FIGURE 1:**
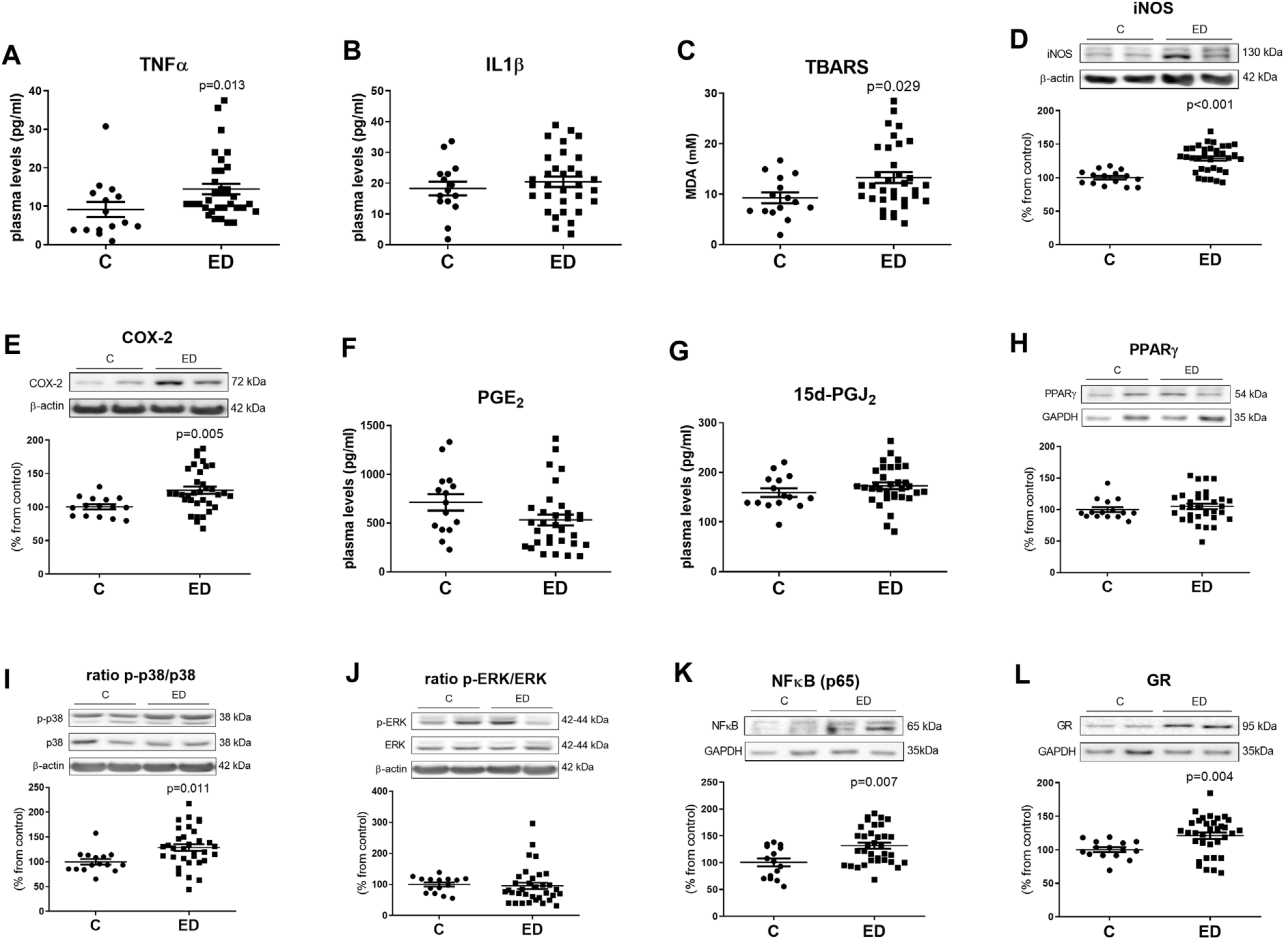
Plasma levels of inflammatory cytokines and oxidative/nitrosative markers **(A–C)**, protein expression levels of inflammatory and oxidative/nitrosative enzymes in the cytosolic fraction of PBMCs **(D,E)**, plasma levels of prostaglandins **(F,G)**, protein expression levels of inflammatory MAPKs in the cytosolic fraction of PBMCs **(I,J)** and protein expression levels of transcription factors **(H,K)** involved in inflammation or receptors involved in the stress response **(L)** in the nuclear fraction of PBMCs. Data are expressed as mean ± SEM on TNFα **(A)**, IL1β **(B)**, TBARS **(C)**, iNOS **(D)**, COX-2 **(E)**, PGE_2_ **(F)**, 15d-PGJ_2_ **(G)**, PPARγ **(H)**, p-p38/p38 **(I)** and p-ERK/ERK ratio **(J)**, NFκBp65 **(K)** and GR **(L)** between eating disorders (ED) and control **(C)** groups (ED *n* = 34, C *n* = 15). In the Western blots the densitometric data of the respective band of interest were normalized by β-actin in the cytosolic extract or by GAPDH in the nuclear extract (lower bands). Data were analyzed using the D’Agostino and Pearson normality test to assess Gaussian distribution, followed by an unpaired two-tailed *t* test. When data did not follow a Gaussian distribution, a Mann-Whitney test was performed; a *p* < 0.05 was considered statistically significant.

Plasma levels of TNF-α (Figure 1A) were higher in the group of patients with ED compared with control. The IL-1β levels were not modified in these patients (Figure 1B).

Furthermore, patients with ED shown increased levels of thiobarbituric acid reactive substances (TBARS; a lipid peroxidation marker) (Figure 1C) in the plasma and higher levels of the oxidative/nitrosative enzyme iNOS (Figure 1D) in PBMCs, when compared with control.

### ED Appears to Affect the Protein Expression of COX-2 Without Affecting its Downstream Products

Patients with ED shown higher levels of COX-2 in PBMCs (Figure 1E) compared with controls.

Plasma levels of the PGE_2_ (proinflammatory) and the 15d-PGJ_2_ (anti-inflammatory) did not change in these patients (Figures 1F,G). The nuclear expression of the anti-inflammatory factor, and receptor of 15d-PGJ_2_, the peroxisome proliferator-activated receptor *gamma* (PPARγ) was not altered in PBMCs from patients either (Figure 1H).

### ED Alter the MAPK p38, the NFκB p65 and the GR Protein Expression Levels

The ratio between the activated (phosphorylated) MAPK p38 and its total form augmented in PBMCs from patients with ED (Figure 1I). However, the ratio between the phosphorylated extracellular signal-regulated kinases (ERK) and its total form did not change when controls and patients were compared (Figure 1J).

The pro-inflammatory NFκB p65 subunit (Figure 1K) as well as the glucocorticoid receptor (GR) (Figure 1L) levels were augmented in PBMCs from patients with ED compared to controls.

### Evaluation of Correlations Among Biological Parameters and Psychological Tests

The possible correlations between all the biochemical parameters analyzed in the study and the clinical/psychological test were investigated. Thus, in patients with ED, significant correlations were observed between PGE_2_ as well as 15d-PGJ_2_ and some clinical parameters.

**FIGURE 2:**
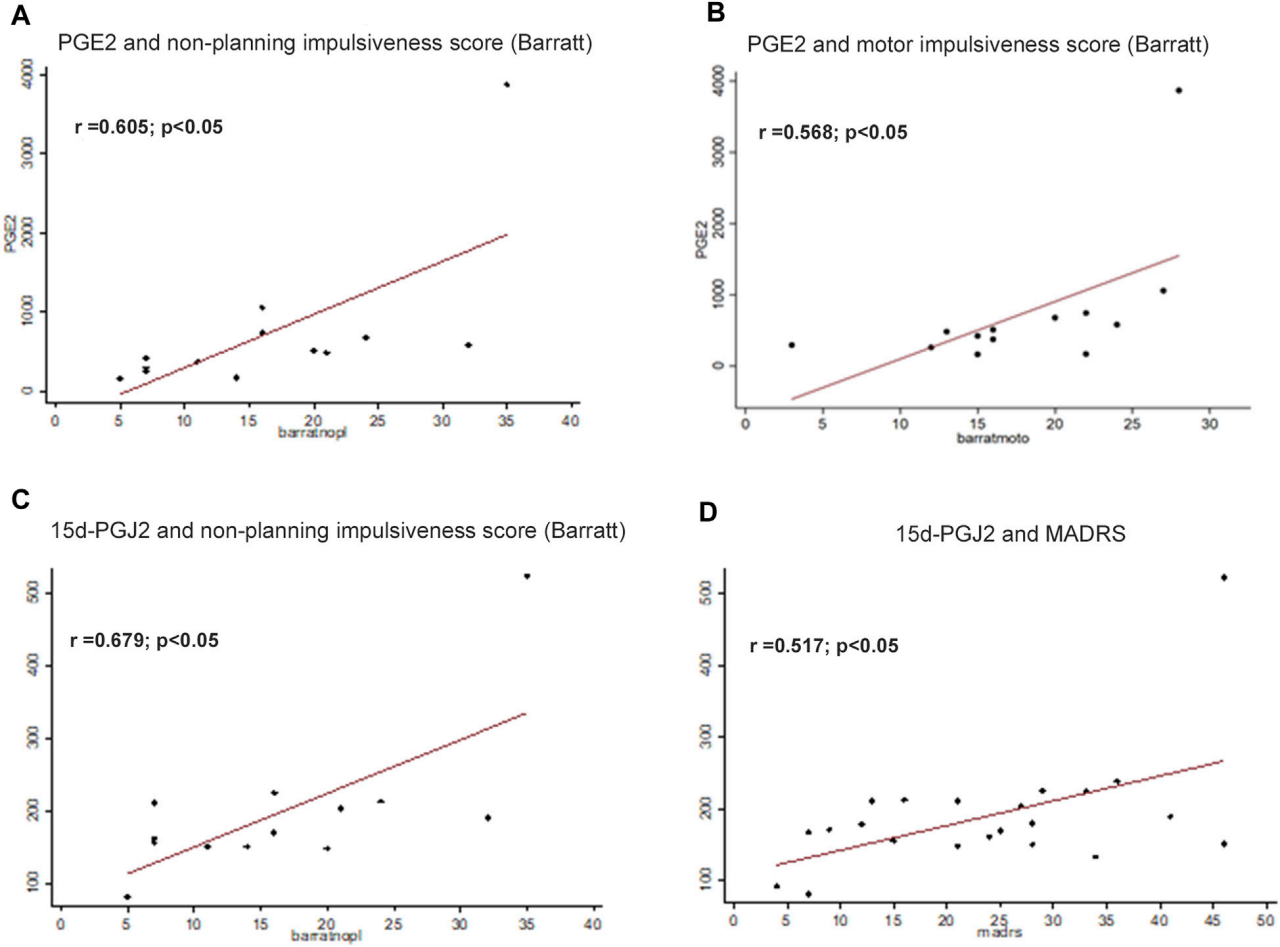
Assessment of correlations among biological parameters and psychological tests. Plasma levels of the inflammatory prostaglandin PGE_2_ were correlated with non-planning impulsiveness score **(A)**, and with motor impulsiveness score (*r* = 0.568; *p* < 0.05) **(B)**. Levels of the anti-inflammatory 15d-PGJ_2_ correlated with Barratt questionnaire non-planning impulsiveness score **(C)** and Montgomery-Asberg Depression Rating Scale (MADRS) score (*r* = 0.517; *p* < 0.05) **(D)**. Correlations were assessed by using Pearson correlation coefficient. The significance was considered *p* < 0.05.

Attending to the Barratt Impulsiveness Scale scores, plasma levels of the inflammatory prostaglandin PGE_2_ correlated with non-planning impulsiveness score (*r* = 0.605; *p* < 0.05) (Figure 2A), and with motor impulsiveness score (*r* = 0.568; *p* < 0.05) (Figure 2B).

Levels of the anti-inflammatory 15d-PGJ_2_ correlated with Barratt questionnaire non-planning impulsiveness score (*r* = 0.679 *p* < 0.05) (Figure 2C) and Montgomery-Asberg Depression Rating Scale (MADRS) score (*r* = 0.517; *p* < 0.05) (Figure 2D).

## Discussion

Our results indicate a relationship between the immune response and impulsiveness and between the immune response and depressive symptomatology in female adult patients with ED. To our knowledge, it is the first time that a relationship between impulsiveness and inflammation in ED has been showed.

The present research shows a dysfunction of the inflammatory/oxidative intra- and intercellular paths in PBMCs and its relationship with impulsivity and affective symptoms in patients with ED. Importantly, none of the patients was under psychopharmacological treatment (excluding benzodiazepines) when the study was conducted.

Patients with ED shown increased levels in the PBMCs of NFκB (a pro-inflammatory nuclear factor) and in the plasma levels of the cytokine TNF-α, an increase in the iNOS expression in PBMCs and a subsequent increment in the oxidative/nitrosative stress indicated by an augmented lipid peroxidation (TBARS assay in plasma). Patients also presented a rise in the COX-2 levels and an increment of the mainly pro-inflammatory MAPK p38 as well as increased expression levels of the glucocorticoid receptor (GR) in their PBMCs.

All the biochemical parameters analyzed were examined for possible correlations with the clinical parameters. The statistical studies indicated that there were significant correlations between some immune response elements and some clinical parameters. In particular, the inflammatory PGE_2_ correlated with motor impulsiveness and with non-planning impulsiveness and the anti-inflammatory 15d-PGJ_2_ correlated with depressive symptomatology as well as non-planning impulsiveness.

In a previous study carried out in a similar sample, it was found an activated immune response in patients with ED (Macdowell et al., 2013). Our current data confirms an increase in the expression of the pro-inflammatory factor NFκB and in the plasma levels of the pro-inflammatory cytokine TNF-α in these patients as well as an oxidative/nitrosative stress. Agreeing with the previous study, patients with ED present an increased COX-2 expression, without changes in prostaglandins levels.

The potential role of pro-inflammatory cytokines in ED has been previously suggested as data from several studies indicate that they have direct and indirect effects on the CNS involved in eating behavior (Corcos et al., 2003). Actually, TNF-α affects the hypothalamic neurons involved in the control of appetite and eating behavior (Konsman and Dantzer, 2001), modifies the firing rate of glucose-sensitive neurons in the lateral hypothalamus (Plata-Salaman, 1998) and it has an impact on neuropeptide-neurotransmitter interfaces (Brambilla et al., 2001).

It is well established that stress is related with mental disorders, including ED. Therefore, it is plausible to formulate a model based on the biological and clinical correlation between stress, anxiety, and ED (Holden and Pakula, 1999) in which the induction of an immune response, resulting in the release of pro-inflammatory cytokines, could be caused by stress: the biological response common to each of these disorders.

Cytokines can induce a NFκB pathway stimulation that it could be also responsible of the increased COX-2 and iNOS protein expression levels, as these enzymes are induced by this nuclear factor (Perkins, 2000). Furthermore, the increased expression of iNOS would explain, at least in part, the oxidative/nitrosative stress present in patients with ED.

The number of articles reporting increased expression levels of COX-2 in PBMCs from patients with ED is extremely limited (Macdowell et al., 2013). This increase, together with the results already mentioned indicates, once again, an inflammatory and oxidative/nitrosative status in this clinical setting. Our data is also showing no differences between patients and healthy controls in the plasma levels of the PGE_2_ and 15d-PGJ_2_ (products of COX-2). A potential explanation could be the moment and the immune scenario at the time of the blood extraction. Another possibility, as it has been described in a previous study employing similar samples, could be the compensatory effect of the cholinergic anti-inflammatory pathway that is being activated in these patients (Macdowell et al., 2013). In any case, more studies are warranted to fully characterize the role of COX-2 in ED.

Regarding the glucocorticoid receptor (GR) our results show an increased expression in PBMCs from patients with ED. Glucocorticoids participate in the modulation of inflammatory processes and an elevation of their levels could translate into a dysregulation of the immune response (Sorrells et al., 2009). Moreover, preceding studies show an anomalous cortisol suppression to dexamethasone test in patients with ED (Diaz-Marsa et al., 2008). Consequently, and although more studies are necessary, it appears like there is an alteration of the hypothalamic-pituitary-adrenal (HPA) axis and a dysfunction in stress management in these patients.

Our work also focused on the psychopathology of ED employing different evaluation tools. In this sense, impulsivity was found to differentiate patients with ED from controls and it has been shown to consistently predict negative outcomes for these patients (Waxman, 2009).

Our data revealed a relationship between impulsiveness and inflammation, as shown by the correlation between the Barratt Scores (motor impulsiveness and non-planning impulsiveness) and the PGE_2_ plasma levels, as well as between the 15d-PGJ_2_ levels and the non-planning impulsiveness Barratt scores.

Stress dysregulation and impulsive personality disorders are intimately associated, and patients frequently exhibit neurobiological stress response dysfunctions and aroused plasma levels of glucocorticoids (Grossman et al., 2003; Diaz-Marsa et al., 2008). Hence, it could be possible to contemplate GR as a sign of an endophenotype with aggressive/impulsive features (Yehuda and Seckl, 2011). This assumption would increase the potential impact of our results involving the GR in patients with ED, although further research is necessary to characterize the possible role of GR as a marker in this clinical setting.

Besides impulsiveness, affective symptomatology has been related to ED; severe depressive symptoms have been associated to further development of ED and to worse ED symptomatology (Evans et al., 2017; Smith et al., 2018).

In our study, a correlation between depressive symptomatology and levels of 15d-PGJ_2_ was found, indicating that there is a relationship between depressive symptoms and the anti-inflammatory pathways. However, no correlation between general anxiety and inflammation was established. Inflammation can be related with depression and anxiety, which often show comorbidity with ED (Hughes et al., 2013). According to our results, depressive symptoms could be mediating between the ED pathology and a dysfunction of inflammatory pathways; this line of research should be pursued in the future to fully comprehend the nature of this correlation.

Our findings need to be weighed in the context of the strengths and limitations of our study. We do believe that a great strength of this work is that none of the patients were taking medication at the time of assessment (for ethics concerns, in emergency cases some of the patients were allowed to take benzodiazepines); this kind of patients, showing great severity of illness and medication-free is quite difficult to recruit. Regarding limitations, this was a study with important ethical issues, as to extract a larger volume of blood from these patients was troublesome. Thus, we were able to study only a limited number of parameters involved in the inflammatory response as the amount of sample was scarce. Another limitation affects the gender of the patients. ED affect people from both genders. However, this is a preliminary observational study, and patients with ED in our population are predominantly females. Thus, we were unable to recruit enough male patients to obtain a sample size with sufficient statistical power and consequently, we decided to focus on female patients. Finally, we lack more information about additional sociodemographic and clinical characteristics of the sample as well as comorbidities with other pathologies, except for the ones included in the exclusion criteria.

In summary, patients with ED without psychopharmacological treatment (excluding benzodiazepines) show an immune activation, displaying increased pro-inflammatory and oxidative/nitrosative stress parameters. From a psychopathological standpoint, plasma levels of the inflammatory prostaglandin PGE_2_ are correlated with motor impulsiveness and with non-planning impulsiveness scores. Additionally, the depressive symptomatology and the non-planning impulsiveness scores are correlated with the levels in plasma of the anti-inflammatory 15d-PGJ_2_.

Thus, inflammatory factors could be considered as potential therapeutic targets in ED, at least as factors to consider in a co-adjuvant treatment of these disorders. Supporting this idea, impulsiveness and depressive symptomatology seem to be linked to an inflammatory dysfunction in patients with ED. Additional studies in this same line of research are required as they could clarify the mechanisms underlying the observed processes offering additional therapeutic strategies.

## Data Availability

The raw data supporting the conclusion of this article will be made available by the authors, without undue reservation.
